# Evaluation of infrared thermography and 6-minute walk tests to assess airflow limitation, impaired thermoregulation, and exercise intolerance in dogs with brachycephalic obstructive airway syndrome

**DOI:** 10.1371/journal.pone.0283807

**Published:** 2023-03-31

**Authors:** Jeremy Gallman, Tekla Lee-Fowler, Stuart Clark-Price, Megan Grobman

**Affiliations:** 1 Department of Clinical Sciences, Auburn University College of Veterinary Medicine, Auburn, Alabama, United States of America; 2 Department of Veterinary Medicine and Surgery, University of Missouri College of Veterinary Medicine, Columbia, Missouri, United States of America; Belgrade University Faculty of Medicine, SERBIA

## Abstract

Brachycephalic obstructive airway syndrome (BOAS) is associated with significant morbidity and mortality. Routine clinical evaluation fails to detect physiologic consequences of BOAS including airflow limitation, exercise intolerance, and impaired thermoregulation. A six-minute walk test (6MWT) with infrared thermography (IRT) may aid detection and clinical management by assessing the physiologic consequences of BOAS. IRT has been used in dogs to assess thermoregulation and in people with obstructive sleep apnea. Our objectives were to compare 6MWT and IRT parameters between healthy mesaticephalic (Mesa) and brachycephalic (Brachy) dogs, and dogs with BOAS. 6MWT parameters include normalized distance walked (ND), rectal temperature, pulse, respiratory rate, and pulse oximetry (SPO2). Mean (T_mean_) and maximum (T_max_) IRT temperatures at 3 regions of interest (ROI) were evaluated. Evaluation timepoints were pre-6MWT, immediately post-6MWT (T_0_) and 5 (T_5_) and 15min post-6MWT (T_15_). No significant difference in ND, SPO2, or temperature were found between groups (p>.05). BOAS dogs had higher dorsal and rostral T_max_ and T_mean_ temperatures compared to Mesa dogs at all timepoints (p < .05). BOAS dogs had higher T_mean_ temperatures compared to Brachy dogs at baseline and T_15_ and T_5_ and T_15_ for dorsal and rostral ROIs respectively (p < .001). ROC analysis showed significant discrimination between BOAS and non-BOAS (Brachy and Mesa) dogs with areas under the curve between 0.79–0.96. Significant moderate correlations were found between IRT temperatures, ND and rectal temperature. This pilot study demonstrates the potential in pairing the 6MWT and IRT with evaluation of clinical signs as screening tool to identify dogs with BOAS.

## Introduction

Brachycephalic obstructive airway syndrome (BOAS) is a clinically significant and potentially life-threatening condition in dogs [1, 2]. Brachycephalic obstructive airway syndrome occurs because of a spectrum of anatomic abnormalities inclusive of, stenotic nares, hyperplastic soft palate, laryngeal collapse, aberrant conchal growth, macroglossia, and hypoplastic trachea [3, 4]. These anatomic changes result in increased upper airway resistance, reduced and turbulent airflow, and upper airway inflammation [3–5]. These predispose affected dogs to aerodigestive and obstructive sleep disorders, and potentially life-threatening upper airway obstruction [6–9]. Furthermore, reduced upper airway airflow inhibits adequate air movement reducing a BOAS-affected dog’s ability to cool themselves through panting [3, 4, 10]. Cumulatively, dogs with BOAS have a shortened lifespan and increased medical costs compared to non-brachycephalic dogs [1, 2]. Early identification and intervention is recommended to reduce long term consequences of BOAS [11].

Currently veterinarians perform physical examinations, thoracic radiographs, and upper airway examinations to identify dogs with BOAS and attempt to assess disease severity [12]. However, in addition to carrying risks associated with sedation and radiation exposure, this assessment is subjective and not linked to physiologic consequences of BOAS: upper airway inflammation and airflow obstruction, hypoxemia, exercise intolerance, and impaired thermoregulation [3, 4, 10, 13]. As such affected dogs may be missed, or disease severity underestimated, when diagnosis is based on anatomic evaluation alone. A 4-point BOAS grading scheme has been developed that incorporates clinical signs with exercise, and whole body plethysmography, in order to identify affected dogs and stratify them by disease severity [14]. This has been adopted as the Respiratory Function Grading (RFG) scheme, to guide better breeding practices in brachycephalic dogs [15].

The six-minute walk test (6MWT), a standardized measure of athletic performance, has been used to evaluate dogs with respiratory disease [13, 16–18]. The 6MWT has been shown to differentiate normal dogs from those with respiratory diseases, as well as differentiate between dogs with mild and severe BOAS using objective endpoint parameters (i.e., distance walked, heart rate, respiratory rate, rectal temperature, and pulse oximetry) [13, 17, 18]. Collectively, these endpoint parameters are used to detect changes in physical performance capacity and facilitate comparisons in endurance between groups. However, these findings have not been correlated with local upper airway inflammation and or airflow obstruction which contribute to the severity and progression of clinical signs.

Infrared thermography (IRT) provides a safe, non-invasive, and radiation-free method for detecting airflow and inflammation by measuring skin surface temperatures [19, 20]. Skin surface temperature correlates with temperatures in deeper tissues [19, 20]. Though not previously used for evaluation of the upper airway in dogs, IRT has been used to evaluate dogs following exercise and to monitor pain and post-surgical inflammation [21–23]. In people, IRT has been used to assess obstructive sleep apnea [24–26]. The presence and severity of obstructive sleep apnea correlates well with IRT surface temperature measurements over the upper airway [25]. English Bulldogs are animal model of obstructive sleep apnea in humans suggesting possible utility IRT for evaluating dogs with BOAS [7].

The objectives of this study were three-fold. Firstly, to evaluate the utility of the 6MWT and IRT to detect changes in physical performance, upper airway obstruction and or inflammation, and impaired thermoregulation in dogs with BOAS (grade 1–3) compared to healthy brachycephalic (grade 0), and healthy mesaticephalic dogs. Secondly, to determine IRT cutoff values discriminating dogs with BOAS from dogs without BOAS. Thirdly, to evaluate results of the 6MWT and IRT between these groups for correlations between upper airway temperature and performance on a 6MWT. We hypothesize that compared to healthy mesaticephalic and brachycephalic dogs, dogs with BOAS will: 1) show evidence of decreased performance capacity on a 6MWT; 2) display higher upper airway temperatures as assessed by IRT; and 3) upper airway temperatures will correlate with distance walked and rectal temperatures.

## Materials and methods

### Case selection and criteria

Dogs presenting to Auburn University Wilford and Kate Bailey Small Animal Teaching Hospital (AU-VTH) between July 20, 2021 and February 25, 2022 were prospectively enrolled into one of three groups (Table 1) [14]. Healthy brachycephalic (Brachy) and mesaticephalic (Mesa) dogs were recruited from companion dogs owned by the staff, students, and faculty of the Bailey Small Animal Teaching Hospital at Auburn University. Dogs with clinical BOAS were recruited from the hospital clinical population.

**Table 1:** BOAS grading scheme.

Group	Head Conformation	Grade	Respiratory Noise	Inspiratory Effort	Dyspnea/Cyanosis/Syncope
Mesa	Mesaticephalic	0	**Pre-6MWT**: None	**Pre-6MWT:** None	**Pre-6MWT:** None
			**Post-6MWT**: None	**Post-6MWT:** None	**Post-6MWT:** None
Brachy	Brachycephalic	0	**Pre-6MWT:** None	**Pre-6MWT**: None	**Pre-6MWT:** None
			**Post-6MWT**: None	**Post-6MWT:** None	**Post-6MWT:** None
BOAS	Brachycephalic	1	**Pre-6MWT:** None-Mild	**Pre-6MWT**: None	**Pre-6MWT:** None
			**Post-6MWT:** Mild	**Post-6MWT**: None-Mild	**Post-6MWT:** None
		2	**Pre-6MWT:** Mild-Moderate	**Pre-6MWT**: Mild-Moderate	**Pre-6MWT:** None
			**Post-6MWT**: Moderate-Severe	**Post-6MWT**: Moderate-Severe	**Post-6MWT:** Mild dyspnea, No cyanosis or syncope
		3	**Pre-6MWT:** Moderate-Severe	**Pre-6MWT:** Moderate-Severe	**Pre-6MWT:** Moderate-Severe dyspnea ± cyanosis, inability to exercise
			**Post-6MWT:** Severe	**Post-6MWT**: Severe	**Post-6MWT:** Severe dyspnea, ± cyanosis or syncope

Adapted brachycephalic obstructive airway syndrome (BOAS) scale [14, 15]. The scale describes the clinical manifestations of upper airway obstruction before and after a six-minute walk test (6MWT). Brachycephalic dogs with a BOAS grade of 0 were enrolled in the Brachy group (i.e., healthy brachycephalic dogs). Brachycephalic dogs with BOAS grade of 1–3 were enrolled in the BOAS group (i.e., clinically affected). Mesaticephalic dogs (Mesa) were also graded to demonstrate lack of clinical evidence of upper airway obstruction (grade 0).

Dogs were included in the Brachy or BOAS groups if they were brachycephalic dogs with BOAS grades 0 and 1–3 respectively (Table 1). Dogs with BOAS were excluded if disease severity was sufficient to prevent safe completion of the study. Dogs were included in the Mesa group if they were a healthy dog with an appropriate craniofacial conformation (mesaticephalic) and no evidence of obstructive upper airway disease. Dogs, regardless group, were excluded from the study if they had previous surgical treatment for BOAS (i.e., stenotic nares, elongated/hyperplastic soft palate, everted laryngeal saccules, laryngeal collapse, or aberrant nasal turbinates), if they had concurrent or historical nasal, respiratory, cardiovascular, or orthopedic disease, or other systemic illness unrelated to BOAS within the preceding 6 months. Dogs were also excluded if their temperament did not allow appropriate image acquisition and measurement of vital parameters (e.g., heart rate, respiratory rate, rectal temperature, and pulse oximetry). Once enrolled, demographic data (age (yrs), breed, sex, body weight (kg), body condition score (BCS; 9-point scale), head conformation (mesaticephalic or brachycephalic), leg length (right front; cm), and duration of clinical signs for BOAS dogs (CS; yrs) were recorded). Leg length was measured from the point of the shoulder to the floor for all dogs. The study protocols were approved by the Auburn University IACUC committee (#2020–3814).

### BOAS grading

The criteria for BOAS grading were adapted from previously published studies and the Respiratory Function Grading scheme (Table 1) [14, 15, 27]. BOAS grading was performed by a single individual (JG). Mesaticephalic dogs were also graded using the same criteria to demonstrate lack of clinical evidence of upper airway obstruction (grade 0).

### Six-minute walk test

A 6MWT was performed as previously described [16–18]. Briefly, the 6MWT was performed along a dedicated, 150 foot-long, temperature-controlled hallway. Dogs were walked with a harness to avoid temperature changes over the cervical region which could be detected by IRT. Rectal temperature (°C), heart rate (bpm), respiratory rate (brpm), and pulse oximetry (%) data were collected at baseline (pre-6MWT), immediately following (T_0_), and at 5min- (T_5_), and 15min-post 6MWT (T_15_). Dogs were allowed to set their own pace and rest if desired. The distance walked was measured (m) and normalized according to leg length.

### Infrared thermographic imaging

**Fig 1:**
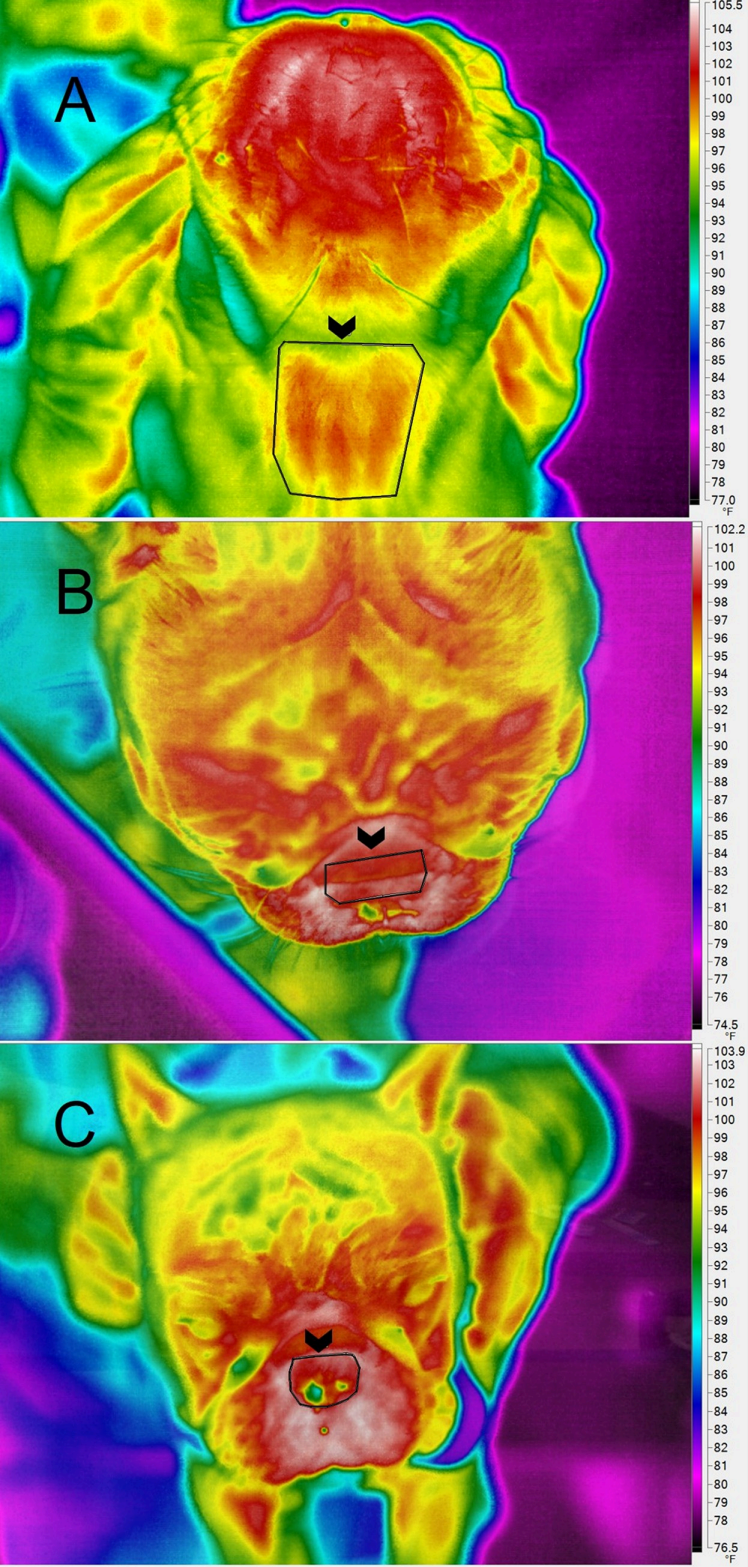
A-C: Infrared thermographic regions of interest (ROI). Infrared thermographic images depicting ROIs which are outlined in black (arrowhead). A) The ventral ROI was drawn to overly the larynx by centering over the ventral neck between the thoracic inlet and the ramus of the mandible. B) The dorsal ROI was drawn over the bridge of the nose between the medial canthi and extending rostrally to the start of the nasal planum. (C) The rostral ROI is drawn to include the nose with nares. Thermal scales are provided to the right of the images.

All enrolled dogs were acclimatized to a standardized room in our hospital for 30 minutes prior to image collection. The mean temperature in the acclimatization, and IRT rooms as well as the hallway was maintained at [23.3°C (74°F)]. The doors of the rooms remained closed during the data collection period to reduce temperature fluctuations. The dogs’ muzzles and necks were not handled from the time the acclimation period started until after all data was collected to prevent artifactual increases in skin temperature [19]. Dogs had ventral cervical, dorsal, and rostral IRT images collected at baseline, T_0_, T_5_, and T_15_ [25]. A standardized region of interest (ROI) was drawn over each image by a single investigator (JG; Fig 1). The mean temperatures (T_mean_) and maximum temperatures (T_max_) within the ROI were calculated and reported. The T_mean_ calculates the average temperature of all the pixels within the ROI. The T_max_ reports the highest temperature pixel within the ROI. For IRT image collection, a Fluke TiX580 camera with a 640 x 480 pixel detector and spectral range of 7.5μm– 14μm was used. The camera was positioned 1.5–2 feet away from the dogs’ necks to standardize distance from the imaged area. An emissivity value of 0.95 was used [19]. Fluke SmartView version 4.3.154.0 software was utilized to analyze the IRT images.

### Statistical analysis

Statistical analysis was completed using commercial statistical analysis software (Sigma Plot 14.5). Descriptive statistics were applied where appropriate. Normality assessment was performed by the Shapiro-Wilks test. Between group comparisons were made using an ANOVA on RANKS and One-way ANOVA and for non-parametric and parametric data respectively. Non-parametric data are presented as median (IQR) and parametric data as mean ± stdev. Receiver operating characteristic (ROC) curves were used to assess the utility of IRT to discriminate the BOAS group from the non-BOAS groups (i.e., Brachy and Mesa). Spearman rank-order or Pearson correlation test was used to evaluate the association between 6MWT and IRT parameters and between T_mean_ and T_max_ temperatures for each ROI and time point. Extrapolating from data in people, a sample size of 10 dogs per group was calculated to detect a 1°C and 20% difference between groups for IRT and 6MWT respectively using a power of 0.80 and significance level of < .05 [25].

## Results

### Animals

Thirty-six dogs were prospectively enrolled: Mesa (n = 16), Brachy (n = 10), and BOAS (n = 10). Sixteen breeds were represented (Table 2). Eighteen dogs were male (2 intact; 16 castrated) and 18 were female (2 intact; 16 spayed). There were no significant differences between groups for age, weight, body condition score, or leg length (*p* ≥.05). Demographic data by group are provided in Table 3. BOAS grades were as follows: Mesa (grade 0; n = 16), Brachy (grade 0; n = 10), and BOAS (grade 1 (n = 6); grade 2 (n = 3) grade 3 (n = 1)). For BOAS dogs, breeds and corresponding BOAS score has been provided in Table 4. For BOAS dogs the median (IQR) duration of clinical signs was 4.88 yrs (1.69–8.50 yrs) (range 1.17–11.8 yrs). Stertor was identified in 10/10 dogs. Stridor was identified in 3/10 BOAS dogs: grade 1 (n = 2) and grade 2 (n = 1). No dog had a history of syncope or had any syncopal events during data collection. All dogs (n = 36) completed the 6MWT successfully completed the study. All dogs were companion animals and per owners did not routinely engage in exercise beyond leash walks.

**Table 2:** Breeds evaluated.

Dogs (n)	Breed
5	French bulldog, Chihuahua mix
4	English bulldog
3	Pit Bull, Brussels griffon, mixed breed dog
2	Boston terrier, Pug, Great Pyrenees
1	Boxer, pit bull mix, Labrador retriever, terrier mix, boxer mix, golden retriever, dachshund mix

Number (n) of dogs of various breeds undergoing six-minute walk test (6MWT) and infrared thermography (IRT).

**Table 3:** Demographic and 6MWT parameters.

Parameter	Mesa	Brachy	BOAS	P value
Age (yrs)	3.5 (2.6–7.5)	7.8 (2.7–10.2)	4.9 (1.7–8.5)	.68
Weight (kg)	25.0 (7.0–31.9)	8.3 (5.3–18.6)	14.1 (10.1–17.5)	.18
BCS (1–9)	5 (4–5.25)	5 (5–6)	6 (5–7.5)	.29
BOAS grade (0–3)	0 (0–0)	0 (0–0)	1 (1–2)	
Leg length (Right Front; cm)	34.3 ± 12.1	27.3 ± 11.5	23.6 ± 3.9	.13
6MWT Distance (m)	420.4 ± 89.3	461.0 ± 60.2	379.8 ± 97.6	.05
Temp (°C)				
Baseline	38.6 ± 0.43	38.9 ± 0.40	38.6 ± 0.70	.22
T_0_	38.7 ± 0.40	39.1 ± 0.40	38.8 ± 0.70	.10
T_5_	38.8 ± 0.40	38.9 ± 0.60	39.0 ± 0.50	.51
T_15_	38.7 ± 0.37	38.9 ± 0.30	38.7 ± 0.60	.91
Heart Rate (bpm)				
Baseline	136.0 (101.0–141.0)	144 (126–147)	140.8 (128–151)	.08
T_0_	109.4 ± 19.3^a,b^	141.6 ± 14.0^b^	145.6 ± 22.5^a^	< .001*
T_5_	106.0 ± 18.0^a,b^	133.8 ± 20.4^b^	138.8 ± 33.2^a^	.002*
T_15_	100.5 ± 20.6^a^	122.6 ± 26.3	131.8 ± 30.5^a^	.01*
Respiratory Rate (brpm)				
Baseline	36 (27–37)	32 (24–40)	28 (24–32)	
	n = 8/16	n = 5/10	n = 5/10	
T_0_	40 (29–54)	44 (34–54)	36	
	n = 6/16	n = 3/10	(n = 1/10)	
T_5_	28 (24–44)	38	32	
	n = 5/16	(n = 1/10)	n = 1/10	
T_15_	32 (22–46)	36 32–36	32 (24–34)	
	n = 7/16	n = 5/10	n = 3/10	
SPO2 (%)				
Baseline	97.5 (96.8–100)	97.5 (97–99)	98.5 (95.3–100)	.48
T_0_	97.5 (96–99)	97.5 (97–99.8)	97.5 (95.3–98.8)	.62
T_5_	98.5 (95.8–100)	100 (97.5–100)	97.5 (94.3–98.8)	.16
T_15_	99 (96–100)	98.5 (96.3–99)	99 (96–100)	.90

Demographic and 6-Minute walk test (6MWT) data are displayed as median (IQR) or mean ± stdev for non-normal and normally distributed data respectively. Statistically significant differences between groups (*p* <0.05) are denoted by an asterisk (*). Due to panting, too few dogs had discrete respiratory rates to allow for statistical comparisons. Median (IQR) or mean ± stdev are provided where able. The number of dogs (n) with recorded respiratory rates out of the group total are provided. The remaining dogs were panting. 6-minute walk test (6MWT), immediately following 6MWT (T_0_), 5 minutes post-6MWT (T_5_), 15 minutes post-6MW (T_15_), brachycephalic (Brachy), brachycephalic obstructive airway syndrome (BOAS), beats per minute (bpm), breaths per minute (brpm), mesaticephalic (Mesa), oxygen saturation (SPO2), years (yrs).

a: groups are statistically different.

b: groups are statistically different.

**Table 4:** Breed and BOAS score in dogs with clinical BOAS.

Breed (n)	BOAS score
Boston Terrier (1)	1
French Bulldog (4)	1,1,1,2
English Bulldog (3)	1,2,3
Pug (2)	1,2

Breeds and BOAS scores for dogs with clinical BOAS (scores 1–3). All dogs in the Mesa and Brachy groups had a score of zero.

### 6-minute walk test

6-minute walk test data by group are provided in Table 3. No significant differences between groups were detected for temperature, SPO2, or normalized distance walked (*p* ≥.05). Heart rates were statistically higher in Brachy and BOAS dogs compared to Mesa dogs at T_0_ and T_5_ and between BOAS and Mesa dogs at T_15_. No significant differences were detected for heart rate at baseline.

### Infrared thermography

Significant differences in T_mean_ and T_max_ temperatures between groups were detected for dorsal and rostral ROIs across all time points (Table 5). No significant differences were detected for ventral ROIs between groups at any time point (*p*>.05). Dorsal and rostral T_mean_ and T_max_ showed significant strongly positive correlations across all time points and the ventral ROI T_mean_ and T_max_ showed significant moderate-strong positive correlations (Table 6). Receiver operating characteristic curves evaluating the dorsal and rostral T_mean_ and T_max_ between BOAS and non-BOAS (Brachy and Mesa) dogs is provided in Table 7. Temperature cut-offs were selected to optimize sensitivity and specificity. Areas under the curve for the dorsal ROI ranged from 0.85–0.94 (T_mean_) and 0.79–0.91 (T_max_). The rostral ROI AUCs ranged from 0.76–0.88 (T_mean_) and 0.73–0.96 (T_max_). Dorsal T_max_ and T_avg_ was not available for 1 dog (BOAS) at T_15_ because the camera angle did not permit accurate drawing of the ROI. Ventral T_max_ and T_avg_ measurements were not available for 1 dog each at T_0_, T_5_, T_15_ respectively. In this case all dogs belonged to the Mesa group. This is because the harness obscured the ROI. All other images and measurements were recorded uneventfully.

**Table 5:** IRT T_max_ and T_mean_ for ventral, dorsal, and rostral ROIs.

	Mesa	Brachy	BOAS	P value
T_mean_	°C			
Ventral				
Baseline	33.1 ± 1.8	33.3 ± 1.7	33.9 ± 1.7	.52
T_0_	32.3 ± 2.6	32.7 ± 2.0	34.1 ± 1.8	.17
T_5_	32.9 (31.5–35.2)	32.9 (31.6–33.3)	34.7 (32.8–35.7)	.22
T_15_	33.1 (31.7–35.3)	33.3 (31.4–34.0)	33.9 (32.7–35.2)	.54
Dorsal				
Baseline	30.47 ± 1.8^a^	31.7 ± 2.5^b^	35.1 ± 3.2^a^^,^^b^	< .001*
T_0_	34.4 (32.1–35.0)^a^	35.3 (33.8–36.2)	37.3 (36.4–37.7)^a^	.002*
T_5_	33.6 (31.8–34.0)^a^	34.7 (36.0–36.4)	37.2 (36.4–37.8)^a^	< .001*
T_15_	32.8 ± 1.2^a^	33.6 ± 2.1^b^	37.0 ± 1.1^a^^,^^b^	< .001*
Rostral				
Baseline	24.9 (23.9–26.6)^a^	25.4 (24.1–27.7.9)	30.1 (26.2–35.0)^a^	.045*
T_0_	28.6 ± 3.6^a^	30.8 ± 2.6	33.5 ± 3.9^a^	.004*
T_5_	27.9 ± 3.2^a^	30.3 ± 2.5^b^	33.8 ± 3.3^a^^,^^b^	< .001*
T_15_	27.3 ± 3.4^a^	29.7 ± 3.1^b^	33.5 ± 3.4^a^^,^^b^	< .001*
T_max_				
Ventral				
Baseline	35.5 ± 1.4	36.6 ± 1.7	36.7 ± 1.2	.08
T_0_	35.2 ± 1.6	36.6 ± 1.6	36.1 ± 1.1	.05
T_5_	35.8 ± 1.4	36.6 ± 1.5	36.8 ± 1.0	.12
T_15_	36.1 ± 1.6	36.8 ± 1.4	36.6 ± 1.8	.51
Dorsal				
Baseline	34.2 ± 1.9 ^a^	35.3 ± 2.4	37.2 ± 2.2^a^	.005*
T_0_	36.4 ± 1.3 ^a^	37.3 ± 1.7	38.2 ± 2.1 ^a^	.007*
T_5_	35.9 ± 1.1 ^a^	36.8 ± 1.1	38.1 ± 1.7 ^a^	< .001*
T_15_	35.6 (35.0–36.5) ^a^	36.6 (36.4–37.2)^b^	38.4 (37.8–39.3) ^a^^,^^b^	< .001*
Rostral				
Baseline	29.6 ± 2.5^a^	32.0 ± 2.8	34.3 ± 4.8^a^	.025*
T_0_	32.9 ± 3.1^a^	35.5 ± 1.5	36.7 ± 3.4^a^	< .001*
T_5_	32.2 ± 3.0^a^	35.0 ± 1.0	37.1 ± 2.0^a^	< .001*
T_15_	32.1 ± 2.8^a^	34.8 ± 2.0	36.7 ± 2.8^a^	< .001*

Infrared thermography data are displayed at median (IQR) or mean ± stdev for non-normal and normally distributed data respectively. Data are displayed in ° Celsius. Statistically significant differences between groups (*p* <0.05) are denoted by an asterisk (*). 6-minute walk test (6MWT), immediately following 6MWT (T_0_), 5 minutes post-6MWT (T_5_), 15 minutes post-6MW (T_15_), brachycephalic (Bracy), brachycephalic obstructive airway syndrome (BOAS), mesaticephalic (Mesa), mean temperature (T_mean_), maximum temperature (T_max_).

a: groups are statistically different.

b: groups are statistically different.

**Table 6:** Correlations between IRT T_mean_ and T_max_.

Correlations	r	P values
Baseline		
Rostral (T_mean_ vs T_max_)	0.90	<0.001*
Dorsal (T_mean_ vs T_max_)	0.90	<0.001*
Ventral (T_mean_ vs T_max_)	0.70	<0.001*
T_0_		
Rostral (T_mean_ vs T_max_)	0.93	<0.001*
Dorsal (T_mean_ vs T_max_)	0.88	<0.001*
Ventral (T_mean_ vs T_max_)	0.77	<0.001*
T5		
Rostral (T_mean_ vs T_max_)	0.85	<0.001*
Dorsal (T_mean_ vs T_max_)	0.87	<0.001*
Ventral (T_mean_ vs T_max_)	0.54	<0.001*
T15		
Rostral (T_mean_ vs T_max_)	0.88	<0.001*
Dorsal (T_mean_ vs T_max_)	0.85	<0.001*
Ventral (T_mean_ vs T_max_)	0.76	<0.001*

Pearson correlation coefficients and p values are displayed for T_mean_ vs T_max_ IRT readings for each ROI. Dorsal and rostral ROIs showed significant strongly positive correlations between T_mean_ and T_max_. The ventral ROI showed significant moderate to strong positive correlations between T_mean_ and T_max_. A p < .05 is considered significant (*). 6-minute walk test (6MWT), immediately following 6MWT (T_0_), 5 minutes post-6MWT (T_5_), 15 minutes post-6MW (T_15_), mean temperature (T_mean_), maximum temperature (T_max_).

**Table 7:** Receiver operating characteristic analysis of BOAS and non-BOAS dogs.

Receiver Operating Characteristic Analysis										
		AUC	Cutoff (>°C)	SE	95% CI	SP	95% CI	LR+	LR-	P value
Dorsal										
T_mean_	Baseline	0.85	35.1	0.70	0.35–0.93	1.0	0.87–1	-	0.3	.001
	T0	0.85	35.7	0.90	0.56–1	0.80	0.61–0.93	4.7	0.12	.001
	T5	0.90	35.6	0.88	0.56–1	0.90	0.7–0.98	7.8	0.11	.0003
	T15	0.94	35.3	1.0	0.66–1	0.92	0.75–0.99	13.0	0	< .0001
T_max_	Baseline	0.81	35.7	0.80	0.44–0.94	0.73	0.52–0.88	3.7	0.33	.004
	T0	0.79	37.7	0.80	0.44–0.97	0.73	0.52–0.88	3.7	0.33	.0007
	T5	0.86	37.9	0.80	0.44–0.97	0.92	0.8–1	4.8	0.05	.0007
	T15	0.91	37.6	0.88	0.52–1	0.92	0.75–0.99	4.1	0.1	< .0001
Rostral										
T_mean_	Baseline	0.76	27.3	0.7	0.35–0.93	0.81	0.61–0.93	3.6	0.37	.016
	T0	0.84	31.7	0.9	0.56–1	0.85	0.65–0.96	5.9	0.11	.001
	T5	0.88	32.3	0.9	0.56–1	0.92	0.75–1	11.7	0.10	.0003
	T15	0.87	31.8	0.9	0.56–1	0.85	0.65–0.96	5.9	0.11	.0006
T_max_	Baseline	0.73	33.5	0.7	0.35–0.93	0.88	0.7–0.98	6.1	0.34	.03
	T0	0.84	31.2	0.9	0.56–1	0.81	0.61–0.93	4.7	0.12	.002
	T5	0.92	36.4	0.9	0.6–1	1.0	0.9–1	-	0.10	.0001
	T15	0.96	36.8	1.0	0.7–1	0.85	0.7–1	6.5	0	< .0001

Receiver operating characteristic (ROC) curve data for Dorsal and Rostral ROIs (T_max_ and T_mean_) used to discriminate between dogs with clinical BOAS and those without BOAS (Brachy and Mesa). Area under the curve (AUC), Sensitivity (SE), Confidence interval (CI), Specificity (SP), Positive likelihood ratio (LR+), Negative likelihood ratio (LR-). Temperature cutoffs were selected for optimal sensitivity and specificity. 6-minute walk test (6MWT), immediately following 6MWT (T_0_), 5 minutes post-6MWT (T_5_), 15 minutes post-6MW (T_15_), mean temperature (T_mean_), maximum temperature (T_max_).

### Correlations between IRT and 6MWT

No significant correlations were identified between distance walked for either ventral, dorsal or rostral T_mean_ at any time point (*p*≥.05). Significant weakly negative correlations were identified between dorsal T_max_ and normalized distance walked at T_5_ (p = .04; r = -0.337) and T_15_ (*p* = .03; r = -0.363). Significant moderately positive correlations were identified between dorsal T_mean_ and rectal temperatures at baseline (*p* = .005; r = 0.435), T0 (*p* = .003; r = 0.479), and T_5_ (*p* = .003; r = 0.475). Additionally, significant weak to moderately positive correlations were identified between rostral T_mean_ and rectal temperatures at baseline (*p* = .03; r = 0.358), and T_0_ (*p* = .006; r = 0.446). Significant weak-moderately positive correlations were identified between dorsal T_max_ and rectal temperatures at baseline (*p* = .01; r = 0.398), T_0_ (*p* = .002; r = 0.494) and T_5_ (*p* = .04; r = 0.334). Significant moderately positive correlations were identified between rostral T_max_ and rectal temperatures at baseline (*p* = .002; r = 0.484) and T_0_ (*p* < .001; r = 0.539).

## Discussion

In this pilot study, the combination of a 6MWT and IRT detected evidence suggesting decreased physical performance capacity (i.e., exercise intolerance) and increased dorsal and rostral ROI temperatures in dogs with clinical evidence of BOAS compared to healthy brachycephalic and healthy mesaticephalic dogs. Importantly, dorsal and rostral T_mean_ and T_max_ were able to discriminate BOAS dogs from dogs without BOAS (i.e., Brachy and Mesa groups) across multiple time points. Upper respiratory tract temperatures demonstrated moderate positive correlations with rectal temperatures and weak negative correlations with normalized distance walked. This pilot study demonstrates the potential value of utilizing the 6MWT and IRT, paired with evaluation of BOAS-compatible clinical signs, to diagnose BOAS and its physiologic consequences in a non-invasive, low risk, and objective manner.

Brachycephalic dogs are increasingly popular despite well documented problems associated with their conformation [28]. Early identification of affected dogs is recommended to reduce long-term complications [11]. This makes clinical recognition of affected dogs of clear clinical importance. Currently the criterion standard for diagnosis involves a physical and upper airway examination with thoracic radiography to identify the characteristic anatomic changes [3]. In addition to carrying risks associated with sedation and or anesthesia (i.e., upper airway examination), these do not identify the important down-stream physiologic consequences of BOAS [3, 5, 10, 13]. Identifying the pathologic consequences of BOAS may significantly impact management recommendations in addition to promoting early identification of affected dogs through low risk clinical screening.

For our pilot study, dogs were evaluated for CS consistent with BOAS adapted from a previously validated scoring system [8, 14, 15, 27]. This method strongly correlated clinical evidence of BOAS with objective evidence of upper airway obstruction using whole body plethysmography and predicted BOAS status with 94–97% accuracy [13, 14, 16, 27]. Similar grading schemes are used to promote responsible breeding practices in brachycephalic dogs (i.e., Pugs, French Bulldogs, and English Bulldogs) [15]. As such, and for the purposes of this study, group assignments were considered appropriate despite the absence of an upper airway examination. In our study, most dogs were considered to have mild BOAS based clinical grade (grade 1: n = 6). Between group comparisons demonstrated no differences in demographic parameters including BCS and leg length which may influence the results of IRT and the 6MWT [29]. Despite the lack of significant differences in leg length, a normalized distance walked was reported to mitigate even minor differences [16].

The 6MWT allows comparison of physical performance between groups following standardized submaximal exercise [16–18]. In previous studies in dogs, the 6MWT differentiated normal dogs from those with respiratory diseases. Dogs with respiratory disease walked significantly less distance during the prescribed six minutes than did normal dogs [13, 17, 18]. The 6MWT has also been utilized to compare English Bulldogs with severe clinical signs of BOAS with those who were only mildly affected. The more severely affected dogs walked a significantly shorter distance during the 6MWT, and took for long for recovery (i.e., heart rate, respiratory rate, upper respiratory noise, and body temperature returning to baseline) [13]. Though no differences in distance walked were detected between groups, the dogs in our study showed differences in other parameters of physical performance. In our study no differences in heart rate between groups was detected at baseline. However, significant differences were detected between groups following exercise [30]. Interestingly, all groups showed subjective decreased heart rate compared to baseline at T_5_ and T_15_. The Mesa and Brachy but not BOAS groups showed decreased heartrate compared to baseline at T_0._ This initial change in heartrate was attributed to decreased excitement compared to the start of the test. Though the heart rates for all groups apparently decreased, they still showed between group difference. At T_0_ and T_5_ both the BOAS and Brachy groups demonstrated increased heart rates compared to the Mesa group. Interestingly, at T_15_ the BOAS group maintained statistically increased heart rates compared to the Meso group while the Brachy group did not. These differences in markers of endurance may suggest that not only do BOAS dogs have evidence of decreased athletic performance compared to mesaticephalic dogs, but they also take longer to recover after exertion compared to their healthy brachycephalic counterparts. No other differences in 6MWT parameters were identified; respiratory rate, and SPO2. The lack of significant differences between groups for other 6MWT parameters may reflect type 2 error as our population was biased toward less clinically affected dogs. Ultimately, larger longitudinal studies are required before the clinical significance of these finding can be determined.

The English Bulldog is an animal model of obstructive sleep apnea (OSA) in humans which, like BOAS, is characterized by upper respiratory tract inflammation and airflow limitation [5, 12, 25]. In humans, the presence and severity of OSA correlates well with IRT surface temperature measurements [25]. In our study, between group comparisons identified significant differences in T_max_ and T_mean_ for the dorsal and rostral but not ventral ROIs. While no significant differences were detected between the Brachy and Mesa groups at any time point, significant differences were detected between both the BOAS and Mesa and BOAS and Brachy groups. These preliminary findings may suggest that clinical signs of disease, based on our scoring system, were associated with objective markers of inflammation and or airflow limitation on IRT, rather than brachycephalic head conformation alone. The reason for the lack of statistical differences using the ventral ROI is unknown as this is a common site of evaluation on people, however, redundant skin folds and differences in haircoat may be considered as a possible barrier due to increased distance from the airway compared to people [25].

Increased dorsal and rostral T_mean_ and T_max_ was found for the BOAS group compared to the Mesa group at all time points. Baseline dorsal T_mean_ was also higher in the BOAS group compared to the Brachy group. Increased temperatures at baseline may suggest underlying inflammation as in human studies of OSA [25]. However, as dogs rely upon evaporative cooling, increased temperatures may reflect a failure of normal heat transfer and impaired thermoregulation because of obstructed airflow. This may be supported by comparing the upper airway temperatures between the BOAS and Brachy groups after exercise. The baseline rostral T_mean_ and dorsal and rostral T_max_ were not significantly different between the BOAS and Brachy groups. However, the BOAS group had increased post-6MWT temperatures compared to the Brachy group at multiple timepoints (Table 4). The dorsal and rostral ROI temperatures were also correlated with rectal temperature. This may support BOAS as a contributor to impaired thermoregulation however concurrent contribution of inflammation to this finding is unknown as inflammation has also been detected in dogs with BOAS [5]. Additional, larger scale studies in this area are needed. Importantly however, these between group differences were not observed on rectal temperature during 6MWT at any timepoint. Therefore, a combination local (i.e., IRT) and systemic (6MWT) evaluation may be useful to evaluate dogs particularly those with low grade disease. Interestingly, dogs in the Brachy group showed no significant differences in ROI temperatures compared to the Mesa group which may imply that the afore mentioned differences reflect clinical BOAS rather than a brachycephalic head conformation in the absence of disease. Receiver operating characteristic analysis demonstrated good-excellent discrimination between the BOAS group and those without BOAS (i.e., Brachy and Meso groups) for both T_mean_ and T_max_. The findings of this pilot study may suggest that IRT with 6MWT could provide a method to screen at risk dogs to help with early, low risk detection of BOAS.

In keeping with our hypothesis, significant moderately positive correlations were identified between ROI temperatures and rectal temperature. This is in keeping with previous studies where IRT measurements at the extremities (e.g. pinna and eye) correlated with rectal temperatures and, as stated above, may reflect impaired thermoregulation [22]. Additionally, significant weakly negative correlations were found between ROI temperatures and distance walked on 6MWT. The weak correlations between IRT and distance walked may reflect the low-grade clinical signs demonstrated by the dogs in the BOAS group.

In our study both T_max_ and T_mean_ are reported. The T_mean_ is commonly reported in the human and veterinary literature and often selected because it is less affected by measurement noise [22, 23, 31–33]. However, this limits automation as it requires carefully hand drawing ROIs to avoid including unintended pixels. The T_max_ reduces operator error and allows for ROI automation because only the warmest pixels are counted [32]. Consistent with studies in people, in our study T_max_ and T_mean_ were correlated [32, 33]. The T_mean_ and T_max_ were significantly and strongly correlated for dorsal and rostral ROIs and moderately-strongly positively correlated for ventral ROIs [32, 33]. However, studies evaluating true agreement between these two measurements, as well as intra- and inter-observer variability, are lacking in the veterinary literature and it is therefore unknown if one could be performed in leu of the other. As such, for this pilot study it was elected to report both T_mean_ and T_max_ measurements [32, 33]. Additional studies focused on these important clinical practicalities are needed before this technique can be widely adopted.

There are a few limitations to this study. Firstly, this study is in a relatively small number of dogs. Though we met our a-priori sample size calculation, additional larger scale studies are recommended including dogs with more significant clinical signs of BOAS. Further, these results have not been evaluated against more conventional methods of diagnosis (e.g., upper airway examination). Though clinical assessment and scoring of brachycephalic dogs based on CS has been validated in dogs [13, 14], as of yet no conclusions can be drawn between the findings on IRT and specific anatomic abnormalities. As such, dogs would still need to undergo conventional evaluation prior to surgical correction. However, IRT and 6MWT could be used as a low risk, minimally invasive screening tool to identify at risk dogs which may benefit from further evaluation. This could be important in cases of early disease or where clients are reluctant to consider anatomic assessment due to cost, risk, or acceptance of clinical signs of BOAS as “normal” leading to delayed recognition and intervention [34]. This technique may also be useful in evaluating non-surgical interventions such as weight loss. An additional limitation is that despite no statistically significant differences in demographic parameters, dogs were not age-, breed-, or weight-matched between groups. Because of the small sample size of this pilot study such comparisons were not performed and breed associated affects cannot be excluded. The ROIs were also hand-drawn which may inadvertently introduce error. We attempted to mitigate this by allowing only one individual (JG) to perform the ROI tracing. We also included evaluation of T_max_ measurements which are less dependent of consistent drawing of ROIs, though intra-observer variability was not performed. Though the technique was generally easy to perform, the ventral ROI was obscured for 1 time point each in 3 Mesa dogs and the dorsal ROI at 1 timepoint in a BOAS dog. The ventral ROI is not likely to be useful give the limitations in data collection as well as the lack of between group differences and differences in neck folds and haircoat. Though dogs had minimal hair covering for the dorsal ROI differences in skin folds may impact IRT results. However, the rostral ROI was successfully collected in all dogs at all timepoints and is less likely to be affected by haircoat, skin color, and skin thickness (e.g., skin folds). Though further studies are needed, this ROI may be the most practical for clinical use. Finally, while BOAS grading and 6MWT were performed by the same individual (JG) IRT images were collected by multiple investigators (MG, TLF, SCP).

## Conclusion

This pilot study demonstrates the potential utility of pairing the 6MWT and IRT with evaluation of CS as screening tool to identify affected dogs with BOAS. This may aid in early clinical diagnosis and improve clinical recommendations by providing a minimally invasive, safe, and objective method of identification of dogs by pairing clinical signs with the physiologic consequences of BOAS: upper airway inflammation and or airflow limitation, exercise intolerance, and impaired thermoregulation.

## Supporting information

S1 Data IRT data. (XLSX)
